# Deciphering epigenetic control of Notch signaling in persistent pulmonary hypertension of the newborn

**DOI:** 10.1038/s41390-025-04234-3

**Published:** 2025-06-17

**Authors:** Matthew D. Durbin, David G. Tingay, Kok Lim Kua

**Affiliations:** 1 Department of Pediatrics, Herman B Wells Center for Pediatric Research, Indiana University School of Medicine, Indianapolis, IN, USA; 2 Neonatal Research, Murdoch Children’s Research Institute, Parkville, Australia; 3 Department of Paediatrics, University of Melbourne, Melbourne, Australia; 4 Department of Critical Care, University of Melbourne, Melbourne, Australia; 5 Center for Diabetes and Metabolic Disease Research, Indiana University School of Medicine, Indianapolis, IN, USA

The pulmonary vasculature undergoes a remarkable transition at birth. In utero, gas exchange occurs at the placenta, and the pulmonary vascular bed remains constricted to direct oxygenated blood from the placenta to the fetal brain. Upon taking their first breaths, a newborn’s pulmonary resistance drops, allowing blood to rapidly flow to the lungs for gas exchange. Persistent pulmonary hypertension of the newborn (PPHN) is marked by sustained high pulmonary vascular resistance, often triggered by perinatal stress.^1^ PPHN causes deoxygenated blood to bypass the lungs via the patent ductus arteriosus and foramen ovale, leading to severe hypoxia and tissue ischemia. PPHN affects 0.4 to 6.8 per 1000 live births,^1^ with mortality rates of 7.6–10.7% and 25% of survivors experience neurodevelopmental impairment.^1^ Treatment is primarily supportive, including oxygen, mechanical ventilation, nitric oxide, and extracorporeal membrane oxygenation.^1^ Understanding the underlying mechanism of PPHN is key to developing new therapies.

In this edition of *Pediatric Research*, Sati and colleagues investigate whether impaired angiogenesis contributes to the elevated pulmonary vasculature resistance in PPHN, focusing on the role of microRNAs.^2^ PPHN was induced in preterm lamb fetuses by surgically constricting ductus arteriosus flow from 128 to 136 days in utero. Lung microRNA expression was compared between these lambs and sham-operated controls (6 per group). The study found that microRNA-30b-5p (miR-30b-5p) was downregulated in PPHN lambs. Furthermore, they show miR-30b-5p promotes angiogenesis in vitro and may regulate Notch signaling by modulating the ligands DLL4 and JAG1.

PPHN is marked by persistent vasoconstriction, excessive muscularization of pulmonary vessels, impaired angiogenesis, and underdeveloped lung tissue.^1^ Studies investigating the abnormal angiogenesis in PPHN have identified altered AMPK function, increased reactive oxygen species, and disrupted Notch signaling.^3,4^ Notch signaling is a highly conserved pathway, essential for numerous cellular processes and implicated in a range of diseases.^5^ NOTCH1-4 are transmembrane receptors that interact with ligands, including delta-like ligands (DLL) and jagged ligands (JAG), and drive cellular processes through transcriptional regulators, including HEY1 and HES1.^5^ Notch signaling is essential for angiogenesis and lung development,^3^ with DLL4 and JAG1 maintaining a balance necessary for sprouting angiogenesis.^6^ Animal models have implicated abnormal Notch signaling in PPHN,^3^ with upregulated DLL4 and downregulated JAG1 hindering tube formation and cell migration. Genetic and epigenetic regulation—including DNA methylation, histone modification, and microRNAs—influence Notch-driven angiogenesis and may offer new therapeutic targets.^7^

MicroRNAs are small noncoding RNA molecules that disrupt messenger RNA translation and inhibit protein synthesis.^8^ MicroRNAs have garnered attention in recent years for their roles in cellular function, development and disease, as well as their potential in research and clinical application.^8^ Increasingly linked to cancer, developmental disorders, and conditions like PPHN,^8,9^ microRNAs also hold potential for therapy, especially for conditions with limited treatment options.^8^ Studies in PPHN lamb models have identified several differentially expressed microRNAs, highlighting their role in disease pathogenesis and therapeutic potential.^9^

This study focuses on miR-30b-5p, a member of the miR-30 family, encoded by six genes across three chromosomes. MiR-30b-5p plays a role in tissue development and disease, and has previously been studied primarily in the context of cancer.^7^ MiR-30 b-5p drives Notch signaling and downstream processes like cell proliferation, migration, and angiogenesis.^7^ A recent study employed a similar lamb model of PPHN, induced by in utero patent ductus arteriosus closure, and demonstrated that miR-30b-5p was downregulated in PPHN pulmonary arterial endothelial cells (PAEC).^9^ The same group also demonstrated disrupted Notch signaling and impaired sprouting angiogenesis in the PPHN lamb PAECs.^3^ The recent study by Sati and colleagues addresses a mechanistic gap, demonstrating miR-30b-5p drives the balance of Notch ligands DLL4 and JAG1, and promotes tube formation, essential for angiogenesis.^2^ These findings highlight a novel epigenetic mechanism of elevated pulmonary pressure in PPHN.

While prior research suggested that PPHN PAECs have downregulated miR-30b-5p and JAG1, and upregulated DLL4, the study by Sati and colleagues provides stronger evidence of this link.^2^ Using RNA in situ hybridization, they show that miR-30b-5p is downregulated, DLL4 is increased and JAG1 is decreased in PPHN. They also show that inhibiting miR-30b-5p reduces tube formation in control PAEC, while angiogenesis, impaired in PPHN PAECs, is restored with a miR-30 mimic. Additionally, HEY1, a downstream Notch1 effector, is increased in PPHN. These findings support a relationship between miR-30b-5p, JAG1, and DLL4 in PPHN pathogenesis.

Although previous studies support miR-30b-5p’s role in promoting angiogenesis, conflicting results left uncertainties.^7,10^ This study clarifies some of those uncertainties, highlighting the nuanced balance of Notch signaling during development. Interestingly, the study found that miR-30b-5p mimic did not affect NOTCH1 or HES1 levels in PPHN, but did increase HEY1 expression. Identifying these signaling pathway characteristics is essential for advancing our understanding and guiding therapeutic strategies.

The study has several limitations that highlight the need for future research. The absence of in vivo data and the small sample size, constrained by the cost and reproductive rate of large animal models, limit generalizability. Lamb models are also subjected to environmental, genetic, and maternal variability. The role of miR-30b-5p in cell types outside of PAEC remains undefined. While the study shows that miR-30b-5p improves angiogenesis, specific effect on downstream targets such as HEY1 and JAG1 are not fully elucidated. Although miR-30b-5p influences JAG1 and DLL4 expression in vitro, direct evidence linking imbalance DLL4/JAG1 to impaired angiogenesis in PPHN is lacking. It is also unclear miR-30b-5p restores angiogenesis through DLL4 regulation. Despite these gaps, the findings support a model in which miR-30b-5p downregulation alters angiogenesis via effects on tube formation and Notch ligands. Future studies should directly test this mechanistic pathway in vivo and across different relevant cell types.

This study is important for researchers and professionals due to the significant burden of PPHN and limited treatment options. Its findings offer deeper insight into PPHN pathogenesis, essential for advancing beyond supportive care. Future research should explore microRNAs both as mechanistic contributors and potential therapies in PPHN. This includes testing in vivo delivery of microRNAs to improve pulmonary vascular resistance and developing targeted delivery systems. While further work is needed to clarify the mechanism, this study identifies a novel epigenetic mechanism in PPHN and marks progress toward understanding and treating this serious condition.
